# Supplementary material for: Inactivation of airborne pathogen surrogates by triethylene glycol
Source: Appl Environ Microbiol. 2026 Jan 23;92(2):e02335-25. doi: 10.1128/aem.02335-25 (PMC12915340; doi:10.1128/aem.02335-25)
Supplement: Supplemental tables — Tables S1 to S3. [file aem.02335-25-s0001.pdf]

Inactivation of Airborne Pathogen Surrogates by Triethylene Glycol

by

Grishma Desai[1], Emanuel Goldman[2], William Jordan[3], Jamie Balarashti[4], Jack Caravanos[5], Rachel Edgar[6], Etienne Grignard[7], Gurumurthy Ramachandran[8], Gediminas Mainelis[9#]

[1] Grignard Company LLC, Rahway, NJ 07065.

[2] Department of Microbiology, Biochemistry, and Molecular Genetics, Rutgers-New Jersey Medical School, Newark, NJ 07103.

[3] Former Deputy Director, Programs, Office of Pesticide Programs, Environmental Protection Agency. William Jordan Consulting, Washington, DC 20016.

[4] President and EOC, Aerosol Research and Engineering Labs, Overland Park, Kansas, 66213.

[5] Clinical Professor of Environmental Public Health Services, New York University, New York, NY 10012.

[6] Department of Infectious Disease, Imperial College London, London SW7 2AZ, UK. Francis Crick Institute, 1 Midland Road, London NW1 1AT, UK.

[7] Founder, CEO, Bleu Garde, LLC, Rahway, NJ 07065.

[8] Department of Environmental Health and Engineering, Johns Hopkins Education and Research Center for Occupational Safety and Health, Bloomberg School of Public Health, Whiting School of Engineering, The Johns Hopkins University, Baltimore, MD 21218.

[9] Department of Environmental Sciences, School of Environmental and Biological Sciences, Rutgers, The State University of NJ, New Brunswick, NJ 08901.

# Address correspondence to Gediminas Mainelis, mainelis@envsci.rutgers.edu

SUPPLEMENTAL MATERIAL

Table S1. Data from exposure of airborne microbes to aerosolized TEG as provided by GrignardPure™ (50% TEG). Data are presented in Figs. 2 and 3.

Time Point	Temp (°C)	RH (%)	Test Device	Microbe	Microbe Type	Avg. TEG conc. (mg/m3)	Control Concentration (cfu/m3)	Control LOG Reduction	Trial Concentrations (cfu/m3)	Trial LOG Reduction	Net LOG Reduction	Error (log10)
10	23.2	37.7	Aura	Phi 6	Enveloped	0.51	6.40E+05 +/- 1.60E+05	0.00E+00	1.60E+04 +/- 4.23E+03	-1.602	-1.602	0.158
35							9.60E+04 +/- 1.39E+04	-8.24E-01	5.33E+02 +/- 9.24E+02	-3.079	-2.256	0.755
50							2.67E+04 +/- 2.44E+03	-1.38E+00	5.33E+02 +/- 9.24E+02	-3.079	-1.700	0.754
80							1.28E+04 +/- 3.20E+03	-1.70E+00	1.07E+02 +/- 1.85E+02	-3.777	-2.078	0.758
10	22.8	38.8	Aura	*Listeria innocua*	Veg, Gram Positive	0.32	1.81E+07 +/- 2.44E+06	0.00E+00	2.88E+06 +/- 5.77E+05	-0.798	-0.798	0.105
35							7.60E+06 +/- 1.20E+06	-3.77E-01	2.13E+04 +/- 4.03E+03	-2.929	-2.552	0.107
50							5.01E+06 +/- 3.78E+05	-5.58E-01	3.04E+03 +/- 2.26E+02	-3.775	-3.217	0.046
80							3.23E+06 +/- 5.08E+05	-7.48E-01	1.92E+03 +/- 4.53E+02	-3.974	-3.226	0.123
10	22.1	33.1	Aura	Methicillin resistant *Staphylococcus epidermidis* (MRSE)	Veg, Gram Positive	0.37	2.45E+08 +/- 8.28E+07	0.00E+00	1.12E+07 +/- 4.80E+06	-1.340	-1.340	0.237
35							8.19E+07 +/- 1.12E+07	-4.76E-01	4.27E+03 +/- 9.24E+02	-4.759	-4.283	0.111
50							3.04E+07 +/- 1.12E+07	-9.06E-01	1.60E+03 +/- 1.60E+03	-5.185	-4.279	0.463
80							1.12E+07 +/- 3.20E+06	-1.34E+00	3.20E+02 +/- 4.53E+02	-5.884	-4.544	0.627
10	23	32.2	Aura	*Salmonella typhimurium*	Veg, Gram Negative	0.45	5.60E+07 +/- 1.82E+07	0.00E+00	1.76E+07 +/- 4.71E+06	-0.503	-0.503	0.183
35							1.33E+07 +/- 4.03E+06	-6.24E-01	5.95E+04 +/- 7.02E+03	-2.974	-2.349	0.141
50							6.45E+06 +/- 5.62E+05	-9.39E-01	2.24E+03 +/- 1.36E+03	-4.398	-3.459	0.266
80							1.81E+06 +/- 1.85E+05	-1.49E+00	3.20E+02 +/- 0.00E+00	-5.243	-3.753	0.044
10	22.4	35.8	Aura	*Klebsiella aerogenes*	Veg, Gram Negative	0.48	1.20E+06 +/- 2.73E+05	0.00E+00	4.96E+05 +/- 1.52E+05	-0.384	-0.384	0.166
35							4.03E+05 +/- 1.34E+05	-4.74E-01	4.00E+03 +/- 6.28E+03	-2.477	-2.003	0.697
50							2.96E+05 +/- 9.60E+04	-6.08E-01	1.07E+03 +/- 3.70E+02	-3.050	-2.442	0.206
80							1.76E+05 +/- 2.12E+04	-8.34E-01	1.07E+02 +/- 1.85E+02	-4.050	-3.216	0.752
10	23.4	36	Aura	*Pseudomonas fluorescens*	Veg, Gram Negative	1.15	3.79E+05 +/- 6.22E+04	0.00E+00	9.92E+03 +/- 9.05E+02	-1.582	-1.582	0.082
35							2.00E+05 +/- 6.28E+04	-2.78E-01	1.60E+02 +/- 2.26E+02	-3.375	-3.097	0.629
50							7.25E+04 +/- 2.79E+04	-7.18E-01	1.07E+02 +/- 1.85E+02	-3.549	-2.831	0.768
80							1.92E+04 +/- 6.22E+03	-1.30E+00	1.07E+02 +/- 1.85E+02	-3.549	-2.254	0.763
10	23.4	36.5	Aura	MS2	Non-Enveloped	1.20	1.87E+10 +/- 4.87E+09	0.00E+00	4.16E+08 +/- 4.45E+07	-1.653	-1.653	0.122
35							7.31E+09 +/- 2.29E+09	-4.08E-01	1.81E+07 +/- 4.89E+06	-3.014	-2.606	0.179
50							4.19E+09 +/- 9.02E+08	-6.50E-01	4.37E+06 +/- 4.03E+05	-3.631	-2.982	0.102
80							2.00E+09 +/- 4.23E+08	-9.71E-01	1.81E+06 +/- 3.70E+05	-4.014	-3.043	0.128
10	23	36.3	Aura	*Mycobacterium smegmatis*	Veg, Mycobacterium	0.79	6.04E+07 +/- 1.23E+07	0.00E+00	1.38E+07 +/- 2.16E+06	-0.641	-0.641	0.112
35							4.05E+07 +/- 1.85E+06	-1.74E-01	3.47E+04 +/- 1.84E+04	-3.241	-3.067	0.232
50							1.56E+07 +/- 3.09E+06	-5.88E-01	1.17E+04 +/- 3.70E+03	-3.713	-3.125	0.162
80							7.36E+06 +/- 1.11E+06	-9.14E-01	2.13E+03 +/- 3.70E+03	-4.453	-3.538	0.756
10	22.7	36.1	Clearify	*Aspergillus brasiliensis*	Endospore	1.96	4.05E+07 +/- 1.85E+06	0.00E+00	2.72E+07 +/- 3.20E+06	-0.173	-0.173	0.055
35							8.53E+06 +/- 9.24E+05	-6.77E-01	1.39E+06 +/- 1.85E+05	-1.464	-0.788	0.074
50							5.33E+06 +/- 9.24E+05	-8.81E-01	2.03E+05 +/- 4.62E+04	-2.300	-1.419	0.124
80							2.77E+06 +/- 9.24E+04	-1.16E+00	8.72E+04 +/- 1.01E+04	-2.667	-1.502	0.052

Table S2. Data from exposure of surface-bound microbes to liquid TEG as provided by GrignardPure™ (50 % TEG). Data are presented in Figs. 2 and 3.

Time Point	Temp (°C)	Test Device	Microbe	Microbe Type	# coupons (per timepoint)	Control Concentration (cfu/ml)	Control LOG Reduction	Trial Concentrations (cfu/ml)	Trial LOG Reduction	Net LOG Reduction	Net Reduction Error (log10)
0						1.52E+04 +/- 1.44E+04	0.000	1.52E+04 +/- 1.44E+04	0.000	0.000	0.583
30	24	50ml conical	Phi 6	Enveloped	4	1.43E+04 +/- 1.60E+04	-0.027	6.67E+01 +/- 7.61E+01	-2.357	-2.330	0.695
60						7.83E+03 +/- 9.73E+03	-0.287	2.00E+01 +/- 1.52E+01	-2.880	-2.593	0.633
0						1.35E+06 +/- 6.54E+05	0.000	1.35E+06 +/- 6.54E+05	0.000	0.000	0.297
30	22.8	50ml conical	*Listeria innocua*	Veg, Gram Positive	4	2.99E+06 +/- 1.93E+06	0.343	2.48E+05 +/- 1.50E+05	-0.737	-1.080	0.384
60						2.38E+06 +/- 1.27E+06	0.246	2.75E+04 +/- 1.98E+04	-1.692	-1.938	0.388
0						1.64E+05 +/- 5.92E+04	0.000	1.64E+05 +/- 5.92E+04	0.000	0.000	0.223
30	22.1	50ml conical	Methicillin resistant *Staphylococcus epidermidis* (MRSE)	Veg, Gram Positive	4	1.38E+05 +/- 6.28E+04	-0.075	5.35E+04 +/- 2.84E+04	-0.485	-0.410	0.304
60						1.15E+05 +/- 5.06E+04	-0.151	1.19E+04 +/- 3.21E+03	-1.139	-0.988	0.224
0						6.34E+07 +/- 3.25E+07	0.000	6.34E+07 +/- 3.25E+07	0.000	0.000	0.315
30	23	50ml conical	*Salmonella typhimurium*	Veg, Gram Negative	4	4.73E+07 +/- 2.43E+07	-0.128	1.28E+06 +/- 7.68E+05	-1.697	-1.569	0.344
60						4.35E+07 +/- 2.25E+07	-0.164	6.45E+04 +/- 3.80E+04	-2.993	-2.829	0.341
0						3.97E+07 +/- 2.08E+07	0.000	3.97E+07 +/- 2.08E+07	0.000	0.000	0.322
30	22.4	50ml conical	*Klebsiella aerogenes*	Veg, Gram Negative	4	9.27E+06 +/- 5.37E+06	-0.632	1.82E+06 +/- 1.54E+06	-1.340	-0.708	0.446
60						7.99E+06 +/- 4.22E+06	-0.696	6.67E+03 +/- 1.72E+04	-3.775	-3.079	1.144
0						4.78E+07 +/- 2.76E+07	0.000	4.78E+07 +/- 2.76E+07	0.000	0.000	0.355
30	23.4	50ml conical	*Pseudomonas fluorescens*	Veg, Gram Negative	4	3.40E+07 +/- 1.80E+07	-0.149	2.15E+06 +/- 1.14E+06	-1.347	-1.199	0.325
60						3.47E+07 +/- 2.33E+07	-0.139	8.90E+04 +/- 3.18E+04	-2.730	-2.591	0.330
0						2.33E+03 +/- 1.66E+03	0.000	2.33E+03 +/- 1.66E+03	0.000	0.000	0.437
30	23.4	50ml conical	MS2	Non-Enveloped	4	8.83E+03 +/- 7.71E+03	0.578	6.17E+03 +/- 5.75E+03	0.422	-0.156	0.555
60						2.67E+03 +/- 2.33E+03	0.058	1.67E+03 +/- 1.76E+03	-0.146	-0.204	0.596
0						2.47E+04 +/- 1.30E+04	0.000	2.47E+04 +/- 1.30E+04	0.000	0.000	0.325
30	23	50ml conical	*Mycobacterium smegmatis*	Veg, Mycobacterium	4	2.78E+04 +/- 1.46E+04	0.052	1.33E+03 +/- 9.02E+02	-1.268	-1.321	0.372
60						3.83E+04 +/- 2.18E+04	0.191	4.50E+01 +/- 3.30E+01	-2.739	-2.930	0.403
0						5.52E+05 +/- 2.32E+05	0.000	5.52E+05 +/- 2.32E+05	0.000	0.000	0.258
30	22.7	50ml conical	*Aspergillus brasiliensis*	Endospore	4	6.13E+05 +/- 2.56E+05	0.045	5.95E+05 +/- 4.03E+05	0.033	-0.013	0.346
60						6.36E+05 +/- 2.86E+05	0.062	4.12E+05 +/- 2.14E+05	-0.127	-0.189	0.298

Table S3. Data from exposure of surface-bound microbes to aerosolized TEG as provided by GrignardPure™ (50 % TEG). Data are presented in Fig. 5.

Time Point	Temp (°C)	RH (%)	Test Device	Microbe	Microb Type	# carriers (per timepoint)	TEG conc. (mg/m3)	Control Load (cfu/carrier)	Control LOG Reduction	Trial Load (cfu/carrier)	Trial LOG Reduction	Net LOG Reduction	Standard deviation
0	24.2	37.2	Aura	Methicillin Resistant Staphylococcus epidermidis (MRSE)	Veg, Gram Positive	4	0.54	4.73E+05 +/- 1.08E+05	0.000	4.73E+05 +/- 1.08E+05	0.000	0.000	0.000
90								3.89E+05 +/- 1.83E+05	-0.085	2.65E+05 +/- 1.49E+05	-0.253	-0.167	0.090
0	24.2	37.2	Aura	Methicillin Resistant Staphylococcus aureus (MRSA)	Veg, Gram Positive	4	0.54	6.09E+05 +/- 1.27E+05	0.000	6.09E+05 +/- 1.27E+05	0.000	0.000	0.095
90								4.73E+05 +/- 2.34E+05	-0.109	3.82E+05 +/- 1.59E+05	-0.202	-0.140	0.066
0	24.2	37.2	Aura	Klebsiella aerogenes	Veg, Gram Negative	4	0.54	3.33E+05 +/- 1.20E+05	0.000	3.33E+05 +/- 1.20E+05	0.000	0.000	0.210
90								8.99E+04 +/- 5.76E+04	-0.568	3.20E+04 +/- 2.99E+04	-1.017	-0.490	0.210
0	24.2	37.2	Aura	Klebsiella pneumoniae	Veg, Gram Negative	4	0.54	1.60E+06 +/- 8.51E+05	0.000	1.60E+06 +/- 8.51E+05	0.000	0.000	0.087
90								5.59E+05 +/- 1.60E+05	-0.457	1.96E+05 +/- 8.55E+04	-0.911	-0.460	0.087
0	24.2	37.2	Aura	Pseudomonas fluorescens	Veg, Gram Negative	4	0.54	5.95E+07 +/- 1.06E+07	0.000	5.95E+07 +/- 1.06E+07	0.000	0.000	663.000
90								8.07E+06 +/- 5.56E+05	-0.867	5.37E+06 +/- 1.14E+06	-1.044	-0.177	0.066
0	24.2	37.2	Aura	Pseudomonas aeruginosa	Veg, Gram Negative	4	0.54	1.38E+07 +/- 7.57E+06	0.000	1.38E+07 +/- 7.57E+06	0.000	0.000	0.066
90								9.11E+05 +/- 5.00E+05	-1.181	5.49E+05 +/- 2.97E+05	-1.401	-0.220	0.066
